# Teriflunomide – The common drug with underestimated oxygen - Dependent anticancer potential

**DOI:** 10.1016/j.bbrep.2021.101141

**Published:** 2021-09-23

**Authors:** Dagmara Otto-Ślusarczyk, Wojciech Graboń, Magdalena Mielczarek-Puta, Alicja Chrzanowska

**Affiliations:** Chair and Department of Biochemistry, Medical University of Warsaw, 02-097 Warsaw, Banacha 1, Poland

**Keywords:** Tissue normoxia, Hypoxia, DHODH, Leflunomide, Teriflunomide, Colon cancer

## Abstract

Leflunomide (LFN) is a well-known immunomodulatory and anti-inflammatory prodrug of teriflunomide (TFN). Due to pyrimidine synthesis inhibition TFN also exhibits potent anticancer effect. Because, there is the strict coupling between the pyrimidine synthesis and the mitochondrial respiratory chain, the oxygen level could modify the cytostatic TNF effect.

The aim of the study was to evaluate the cytostatic effect of pharmacologically achievable teriflunomide (TFN) concentrations at physiological oxygen levels, i.e. 1% hypoxia and 10% tissue normoxia compared to 21%

oxygen level occurred in routine cell culture environment.

The TFN effect was evaluated using TB, MTT and FITC Annexin tests for human primary (SW480) and metastatic (SW620) colon cancer cell lines at various oxygen levels.

We demonstrated significant differences between proliferation, survival and apoptosis at 1, 10 and 21% oxygen in primary and metastatic colon cancer cell lines (SW480, SW620) under TFN treatment. The cytostatic TFN effect was more pronounced at hypoxia compared to tissue and atmospheric normoxia in both cancer cell lines, however metastatic cells were more resistant to antiproliferative and proapoptotic TFN action. The early apoptosis was predominant in physiological oxygen tension while in atmospheric normoxia the late apoptosis was induced.

Our findings showed that anticancer TFN effect is more strong in physiological oxygen compared to atmospheric normoxia. It suggests that results obtained from *in vitro* studies could be underestimated. Thus, it gives assumption for future comprehensive studies at real oxygen environment involving TNF use in combination with other antitumor agents affecting oxygen-dependent pyrimidine synthesis.

## 1 Introduction

Specific changes in cellular metabolism lead to key processes important for cancer development – uncontrolled growth and proliferation. Majority of studies conducted on cancer focus on glucose metabolism and the glycolytic pathway, however it is known that tumor cells have fully functional mitochondria [1]. The lack of mitochondrial DNA (mtDNA) does not allow for proliferation of tumors cells unless they are able to recover their own oxidative phosphorylation (OXPHOS) by acquisition of stromal mitochondria. Pyrimidine biosynthesis dependent on respiration-linked dihydroorotate dehydrogenase (DHODH) is crucial for cell growth, while mitochondrial ATP generation is not essential for proliferation [2,3]. It seems that pharmacological inhibition of mitochondrial metabolism can be used as a potential therapeutic strategy in some cancers.

As a central metabolic organelle, mitochondria have a critical biochemical functions for the synthesis of basic cellular components, including nucleotides. Among the enzymes of *de novo* pyrimidine biosynthesis pathway only DHODH is localized in mitochondria. Cell demand of both purines and pyrimidines rises during increased proliferation. The integral step of *de novo* pyrimidines synthesis is catalyzed by the flavoenzyme dihydroorotate dehydrogenase (DHODH) which provides oxidative reaction relies on the conversion of dihydroorotate to orotate. Subsequent catalytic steps include conversion of orotate into uridine monophosphates which can be further converted to UTP and CTP, and finally, to dTTP and dCTP. DHODH is located in the mitochondrial inner membrane with the active site facing in and is being functionally connected to the Electron Transport Chain (ETC) by a flavin prosthetic group that couples dihydroorotate oxidation with reduction of ubiquinone to ubiquinol followed by electron flow through the ETC [4]. As a result of the functional association between DHODH and the ETC, it has been suggested that any dysfunction of the ETC i.e. lack of oxygen, presence of inhibitors or mutations of complex III and IV, can cause impairment of *de novo* UMP synthesis and a subsequent decrease in *de novo* pyrimidine synthesis and, thereby, the cytosolic ribonucleotide pool. Considering that DHODH is essential for nucleic acids synthesis, using the inhibitor of this enzyme can decrease cell proliferation [2,5]. A known pharmacological DHODH inhibitor is pro-drug leflunomide which in the hepatic metabolism is converted to its active metabolite – teriflunomide (TFN). Teriflunomide undergoes enterohepatic circulation and biliary recycling resulting in steady state high plasma concentration with retarded elimination half-life, shows mild adverse effects, tolerability profiles and the convenience of oral administration [6]. Leflunomide (LFN) as anti-inflammatory and immunomodulatory drug is commonly used for rheumatoid arthritis treatment. Its immunosuppressive activity consists of suppression of proliferating T- and B-lymphocytes and presumably interleukin–2 (Il-2) [7]. On the other hand, there are many reports about LFN and TFN anticancer potential in several human neoplasms, such as chronic lymphocytic leukemia (CLL) [8], melanoma [9], prostate cancer [10], triple negative breast cancer [11], multiple myeloma [12], and carcinoids [13]. Mechanism of anticancer activity is associated with interferences of cell cycle, inhibition of cancer cell proliferation and induction of cell death [14].

Many studies *in vitro* are carried out using LFN to assess the cytostatic effect of the drug. It should be stressed that LFN is only a prodrug with no own pharmacological activity. In our opinion, results of these studies are unreliable since there are no available information that neoplastic cells are able to convert LFN to TFN.

Therefore, we investigated the antitumor effect of TFN as active agent in pharmacologically achievable concentrations.

As yet all studies of TFN anticancer activity *in vitro* have been conducted at supraphysiological oxygen level i.e. 21%. Taken into account that DHODH action is oxygen – dependent via ETC, we have assumed that lower oxygen level present in tumor milieu could enhance TFN antiproliferative potential *in vivo* while the studies carried out at 21% oxygen may yield underestimated results due to higher oxygen concentration.

Accordingly, in this study for the first time we evaluated the anticancer effect of TFN at tissue normoxia (10%) and hypoxia (1%).

## 2 Materials and methods

### 2.1 Materials

#### 2.1.1 Cell culture conditions and treatments

SW480, SW620 (primary and metastatic colorectal cancer from the same patient) and HaCaT (immortalized human keratinocytes) cells were purchased from the American Type Culture Collection (ATCC) and cultured according to protocol. Medium was supplemented with 10% heat-inactivated fetal bovine serum, penicillin (100 U/ml), streptomycin (100 μg/ml). The cells were cultured until 80–90% confluence was reached, then were harvested by treatment with 0.25% trypsin-0.02% EDTA in phosphate-buffered saline solution (PBS) and used for experiments. Teriflunomide was diluted by pure medium in order to achieve final culture concentration 100 μM and 200 μM. Oxaliplatin was diluted by PBS and a final concentration in culture of 3 μM was achieved. Both malonate and sodium azide solution were prepared using PBS to obtain final culture concentration of 500 μM.

To determine the effect of oxygen and teriflunomide, cells were seeded in 6, 12 or 96-well plates. After 24 h pre-incubation, leflunomide (100 μM) and (200 μM), oxaliplatin, malonate or azide were added. Untreated cells were used as control. Cells were cultured in hypoxic (<1% O_2_), tissue normoxic (10% O_2_) and atmospheric normoxic (21% O_2_) conditions in Hypoxic Chamber with oxygen controller (Coy Laboratory Products INC, USA) for 72h and used for further experiments. Untreated cells were used as the control.

### 2.2 Methods

#### 2.2.1 Trypan blue assays

Cells were seeded on 12-well plates (lx10^5^ cells/well) and cultured at condition mention above. After 72h medium was removed and after washing cells two times with PBS, they were trypsinized. Cell number and viability were assessed using a trypan blue exclusion assay by automated cell counter (Countess Invitrogen).

#### 2.2.2 MTT assay

The cell viability was assessed by using enzymatic conversion of 3-(4,5-dimethylthiazol-2-yl)-2,5-diphenyltetrazolium bromide salt (MTT) to insoluble formazan crystals by mitochondrial dehydrogenases occurring in living cells. Cells were seeded in 96-well plates (lx10^4^ cells/well) and cultured at condition described previously [15]. The relative MTT level (%) was calculated as [A]/[B] ×100, where [A] is the absorbance of the test sample and [B] is the absorbance of control sample containing the untreated cells. Cell viability was presented as a percent of reduced MTT in treated cells versus control. IC50 value was estimated using CompuSyn version 1.0.

#### 2.2.3 FITC Annexin V binding assay

The SW480, SW620 and HaCaT cells were seeded in 6-well plates (2 × 10^5^ cells/well) and cultured under the conditions mentioned at the Cell Culture section. After 24 h pre-incubation cells were treated with IC_50_ concentrations of tested compounds and incubated for 72 hours. The apoptotic effect was determined by dual staining with Annexin V:FITC and propidium iodide (PI) detection kit (FITC:Annexin V Apoptosis Detection Kit I; BD Biosciences Pharmingen) according to manufacturer's protocol and literature data [16] and analyzed by flow cytometry (Becton Dickinson) [15]. Cells which were Annexin V:FITC positive and PI negative were identified as early apoptosis, and Annexin V:FITC and PI positive as late apoptosis or necrosis.

#### 2.2.4 Statistical analysis

The statistical calculation was performed using Statistica 13.1 (StatSoft, Inc, USA) program. Quantitative comparison between studied groups was performed by t-Student test. Data are expressed as the mean ± SD from three independent experiments performed in triplicate and considered statistically significant at p < 0.05.

## 3 Results

### 3.1 Effect of leflunomide, oxaliplatin, malonate and azide on cell number and viability at various oxygen levels

#### 3.1.1 TB assay

**Fig. 1:**
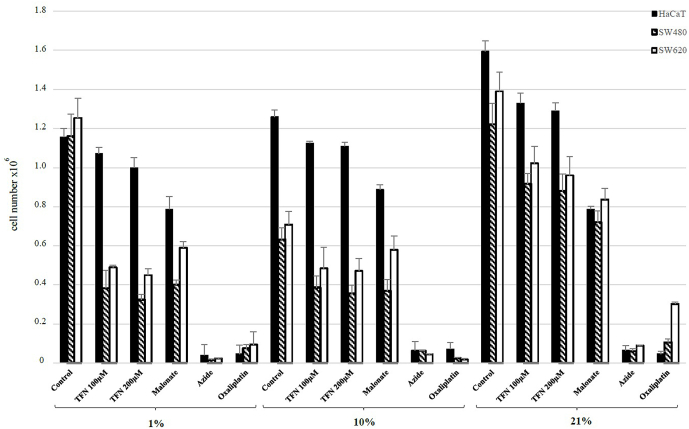
The effect of teriflunomide at concentrations 100 and 200 μM, malonate, azide and oxaliplatin on the number of live cells at various oxygen tensions (1,10 and 21%) using TB exclusion assay. Cells were cultured as indicated in experimental section. Solid bar- HaCaT cells, crossed bar- SW480 cells, open bar – SW620 cells. Results were expressed as means ± SD.

The influence of studied compounds on cell number at hypoxia and both normoxias analyzed by TB method was summarized in Fig. 1. The mean cell number of both SW480 and SW620 cells with TFN (100 μΜ) and TFN (200 μM) was lower than in adjacent controls in all tested oxygen tension, however it didn't exhibited a significant concentration-dependent effect. At 1% oxygen mean cell number of SW480 decreased nearly 3 times as compared to the control (p < 0.001) whereas the decrease of cell number at 10% in the presence of both TFN concentrations wasn't so spectacular compared to hypoxia (above 1.7 times) (p < 0.001). TFN effect at 21% was least pronounced resulting in decrease of the mean SW480 number by only 1.3 times (p < 0.05). The viability of SW480 cells treated with both concentrations TFN in all oxygen tensions was comparable and ranged from 71%-79%. The lowest cell survival was found at hypoxia with average 72.2% ± 0.8.

In the SW620 cells cultured with TFN (130 μΜ) and TFN (260 μM) at 1% oxygen the cell number was more than 2.7 times lower than in control cells (p < 0.001) while at 10% it dropped about 1.5 folds (p < 0.05). At 21% cell number in SW620 treated with TFN (100 μΜ) and TFN (200 μM) was significantly higher than at 1% and 10% (p < 0.001). The viability of SW620 cells treated with both concentrations of TFN in all oxygen tensions was higher than in primary cancer cells and ranged from 78%-82%. The lowest SW620 viability was revealed at hypoxia where it accounted 78.4% ± 0.24.

Addition of uridine in physiological concentration (10 μM) to culture medium didn't protect cancer cells from antiproliferative TFN effect (data not shown).

HaCaT cells were found to be more resistant to teriflunomide effect. The lowest normal cell sensitivity was observed in hypoxia (1.14 ± 0.04 for control vs 1.07 ± 0.03 and 0.99 ±0 .0.05 for TFN 100 and 200 respectively). Both TFN concentrations showed the strongest cytotoxic effect in normal cells at 21% where the cell number decreased 1.2 times. However, the viability was high and reached a range of 92–95%.

To assess the inhibition of TCA cycle and ETC, well-known inhibitors: malonate and azide were used.

Antiproliferative effect of malonate on both cancer cell lines was similar at 1% and 10% whereas at 21% was less pronounced. Both azide and oxaliplatin were highly cytotoxic for cancer and normal cell lines compared to malonate. Cytotoxic effect of azide was highest against SW480 and SW620 cells at hypoxia. The cell number of SW480 was comparable at 10 and 21% in azide presence, whereas in SW620 cells azide was more effective in 10% than 21%. The cell viability after malonate treatment was quite high and accounted from 88% to 93% whereas for azide was lower and accounted from 57% to 62%.

Oxaliplatin showed the strongest effect on proliferation of SW480 and SW620 cells in 10% oxygen tension. In 21% the oxaliplatin less inhibited proliferation, and in metastatic cancer cells especially this effect was much weaker. The cell survival was low and accounted from 58% to 64% in all oxygen tension.

#### 3.1.2 MTT assay

**Fig. 2:**
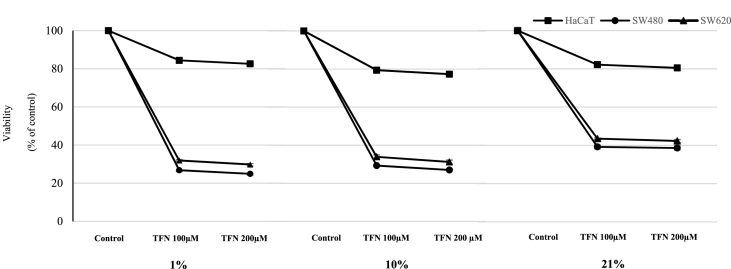
The effect of teriflunomide at concentrations 100 and 200 μM on cell viability at various oxygen tensions (1,10 and 21%) in SW480, SW620 and HaCaT cells using MTT test. Values are expressed as percentage (%) of viable cells with respect to controls. Cells were cultured as indicated in experimental section.

The concentrations 100 and 200 μM of teriflunomide used in the study affect viability of SW480 and SW620 cells considerably (Fig. 2).

The lowest cell count was observed at hypoxia in the presence of both studied TFN concentrations in primary and metastatic cell lines. TFN (100 μΜ) and TFN (200 μM) showed decrease to 26.8% ± 0.4 and 24.9% ± 0.7 in SW480, and 31.9% ± 0.5 and 29.8% ± 0.6 in SW620 respectively (p < 0.001). The number of cancer cells after incubation with TFN (100 μΜ) and TFN (200 μM) was slightly higher at 10% and accounted for 29.3% ± 0.4 and 27% ± 0.4 in primary cancer cell line whereas in SW620 they were 33.95% ± 0.5 and 31.2% ± 0.3 respectively (p < 0.001). The highest count was exhibited by SW480 and SW620 cells in atmospheric normoxia and after treatment with corresponding TFN concentrations for primary cell line was 39.2% ± 0.4 and 38.6% ± 0.7 respectively whereas for metastatic cancer cell line it was 43.5% ± 0.07 and 42.3% ± 0.7 (p < 0.001).

#### 3.1.3 Effect of leflunomide on early and late apoptosis at various oxygen levels

**Fig. 3:**
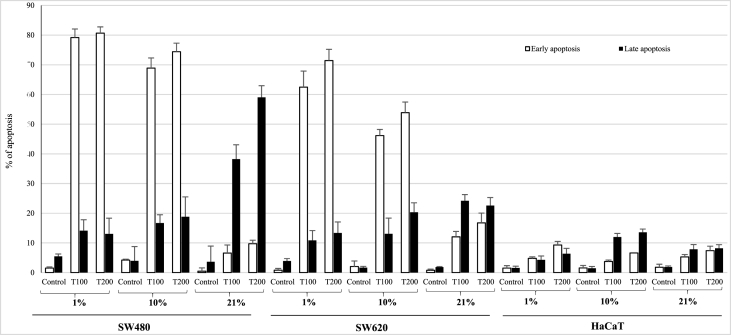
The effect of teriflunomide at concentrations 100 and 200 μM on early and late apoptosis or necrosis at various oxygen tensions (1,10 and 21%) in SW480, SW620 and HaCaT cells detected by flow cytometry. Open bar – early apoptosis, solid bar – late apoptosis. Cells were incubated for 72 with compounds. Data are expressed as % of cells at early stage of apoptosis and % of cells at late stage apoptosis. Data are expressed as means ± SD.

**Fig. 4:**
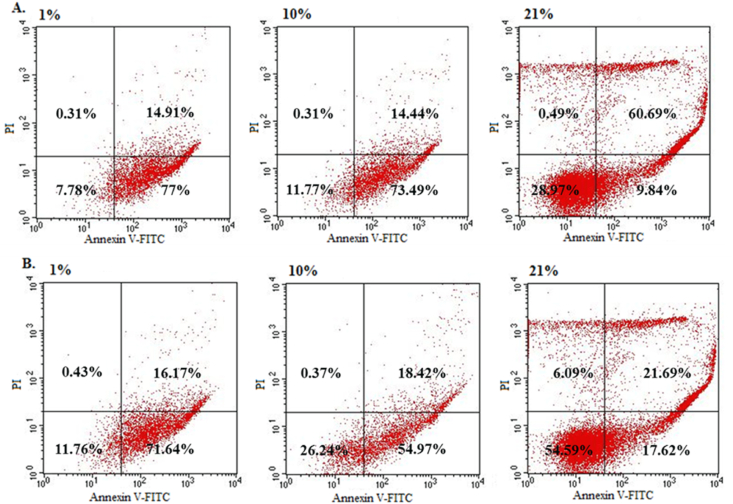
The effect of teriflunomide at concentrations 200 μM on stages of apoptosis or necrosis at various oxygen tensions (1,10 and 21%) in **(A)** SW480 and **(B)** SW620 cells detected by flow cytometry. Cells were incubated for 72 h with compounds. Graphs show representative experiments. The lower right quadrant shows early apoptotic cells. The upper right quadrant represents late stage of apoptosis and upper left quadrant represents necrosis.

To assess the proapoptotic action of teriflunomide *in vitro*, the effect of two concentrations 100 μM and 200 μM on early and late apoptosis using flow cytometry analysis was provided. Results are presented in Fig. 3, Fig. 4. The incubation of SW480 and SW620 cells with both concentrations of TFN (100 and 200 μM) showed highest percentage of cells in early apoptosis at 1% as compared to the controls (79.2% ± 2.9 and 80.7% ± 2.1 vs 2.5% ± 1.4, p < 0.001) and (46.1% ± 2.1 and 53.9% ± 3.6 vs 3.93 ± 0.6, p < 0.001) respectively. The increase of early apoptosis after treatment with both TFN concentrations (100 and 200 μM) was also noticed in 10% in both primary (66.2% ± 3.4 and 75.2% ± 3.1) and metastatic (46.3% ± 2.4 and 53.7% ± 3.5) cancer cell lines. However in SW480 it was more significant (p < 0.001). Additionally in tissue normoxia more cells entered late apoptosis. In atmospheric normoxia (21%) TFN slightly affected the level of early apoptosis in both cancer lines simultaneously inducing extensive late apoptosis. The rise of early apoptosis in this cell line was several times higher whereas level of late apoptosis was dozen times higher than control. After treatment with both TFN concentrations (100 and 200 μΜ) percentage of late apoptotic cells was 38.2% ± 4.8 and 59.1% ± 3.9 vs control where it accounted for 3.6% ± 1.3.

The apoptosis assay performed in HaCaT cells treated with studied compound in both concentrations showed that keratinocytes were not so sensitive to TFN influence as cancerous lines. The highest increase of early apoptosis was observed at tissue hypoxia (1%) in keratinocytes treated with TFN (100 and 200 μΜ). Contrary, the highest rise of late apoptosis was observed in tissue normoxia (10%).

Annexin assay results present in current work corresponded with MTT and TB findings indicating that treatment with both teriflunomide concentrations can induced early or late apoptosis in studied cancer cells.

## 4 Discussion

Until now, the majority of studies conducted on tumor cell lines have been carried out at 21% oxygen level (atmospheric normoxia), while oxygen tension in tumor environment never exceeds 10% (tissue normoxia). Therefore, the data obtained under atmospheric normoxia conditions do not reflect tumor metabolism *in vivo*, especially for oxygen-dependent enzyme activity.

There have been no investigations on the association of leflunomide potency with DHODH at physiological oxygen levels (1% and 10%). It seems to be particularly interesting because of the DHODH link with the mitochondrial ETC.

We found that contrary to normal cells number of primary and metastatic colon cancer cells was higher at 1% hypoxia than at 10% tissue normoxia. From these observations it can be concluded that hypoxic conditions appear to promote increased proliferation of cancer cells but not normal cells. Tumor hypoxia plays a substantial role in selecting cells that can survive the metabolic stress associated with low oxygen tension. Those results confirmed previous observations that hypoxia was not a limiting factor for cancer cells proliferation but are contradictory with obtained by Chen et al. where pyrimidine synthesis was impaired at hypoxia due to suppression of human *cad* gene by HIF-1 [17]. Furthermore, medium supplementation with uridine didn't salvage cancer cells from TFN cytotoxic effect. Similarly, Hail et al. observed no exogenous uridine influence on skin cancer cell growth. It should be mentioned that uridine supplementation can support some cancer cell growth [18]. Considering above findings, it is promising that colon cancer cells growth is dependent solely on endogenous uridine synthesis. Thus, more efficient TFN effect may be expected in cancer patients.

Interestingly, there is report about a lack of DHODH expression in SW480 cells which should result in markedly impaired proliferation [19]. It is inconsistent with our data.

At 21% oxygen the cell number of each studied cells was higher than at both 10% and 1% oxygen, however difference was higher in the former case. It indicates that atmospheric normoxia (21% oxygen), a non-physiological condition, does not reflect the scenario *in vivo.*

An opposite pattern of oxygen effect on cell number was revealed for control cells. The most marked difference between number of cancer and normal cells was observed at 1% where their growth was much less intensive as compared to tissue and atmospheric normoxia.

TFN was shown to be effective in terms of proliferation of both primary and metastatic cancer cell lines. TFN treatment was more pronounced under hypoxic conditions compared to tissue normoxia. This indicates mutual effect potentialization of two factors influencing DHODH activity. In contrast, normal cells were more sensitive to TFN at tissue normoxia. Our results corresponding with findings of Karaman et al. and indicating that TFN use under limited oxygen availability resulted in anti-ischemic effect [20]. However, 21% oxygen attenuated antiproliferative TFN action on cancer but not normal cells. Pharmacologically achievable plasma TFN concentrations (100 and 200 μM) were used in our study [21,22]. We found that TFN activity is not dose-dependent within this concentration range (Fig. 1, Fig. 2). Our findings are consistent with those obtained by Hail et al. indicating equal efficacy of above TFN concentrations [10]. Malonate is a known inhibitor of succinate dehydrogenase (SDH) – the enzyme localized in inner mitochondrial membrane and functionally coupled with ETC. Therefore we used this compound since TFN is an inhibitor of DHODH which localization and function are analogical to SDH. However TFN appeared to be more effective than malonate in hypoxia and tissue normoxia indicating that DHODH inhibition exerts more marked influence on proliferation than TCA cycle and ETC inhibition. DHODH blocking effect by TFN was dependent on oxygen level, whereas malonate exhibited similar antiproliferative activity in physiological oxygen levels (1% and 10%) but weaker in atmospheric normoxia. Studies conducted on cancer cells with SDH mutation showed that succinate accumulation lead to normoxic HIF 1 alfa activity due to prolyl hydroxylase inhibition. It should result in malonate activity potentialization at 10% O_2_ [23]. Interestingly, we didn't observe any difference in malonate influence on proliferation at hypoxia and tissue normoxia.

It suggests that decrease in pyrimidine biosynthesis has a greater impact on cells growth at physiological oxygen levels compared to mitochondrial energetic disturbances.

Additionally, azide was used as a standard ETC inhibitor to mimic metabolism of respiration deficient cells [24]. It exhibited outstanding but non-selective antiproliferative potency for all studied cell lines. Similar to other used inhibitors it exerted less marked effect at atmospheric normoxia. Azide cytotoxic effect was much stronger compared to malonate in all studied oxygen levels.

Oxaliplatin was chosen as a comparative cytotoxic agent used in CRC therapy with distinct ETC independent antiproliferative activity [25,26]*.* Our observations revealed that its effect on cancer cell growth was markedly higher at tissue normoxia as compared to hypoxia. It may be result of oxaliplatin induced greater reactive oxygen species (ROS) generation. Interestingly, metastatic cancer cells proved to be more resistant to oxaliplatin at atmospheric normoxia. It indicates that oxaliplatin cytotoxic effect *in vivo* is underestimated.

MTT assay was performed for TFN but not malonate and azide because both compounds as they are mitochondrial inhibitors, may interfere with MTT results which are based on mitochondrial redox reaction measurement. DHODH doesn't participate in the main ETC route however it could interfere with MTT assay. Indeed, the dead cell number obtained from MTT test was higher compared to TB. Additionally in our study, compatibility between TB and MTT tests was dependent on oxygen tension; the results were closest in hypoxia. It indicates that oxygen level could affect viability determined by mitochondrial metabolism tests such as MTT.

An early apoptosis was a dominant cell death mechanism at physiological oxygen tension (1 and 10%) following TFN treatment in both cancer cell lines, whereas at atmospheric normoxia prevailed a late apoptosis was prevailed.

This indicates a discrepancy between the oxygen level in cells *in vivo* and *in vitro*, which may result in unreliable data. The studies conducted by other researches are usually performed in artificial conditions i.e. atmospheric normoxia 21% O_2_. Therefore our findings may be comparable only with those obtained under the same conditions. There are limited data about TFN induced apoptosis. For CLL (chronic lymphoblastic leukemia) cells, late apoptosis was observed after 96 hours but in suprapharmacological TFN concentrations (80–120 μg/ml i.e. approx. 300–450 μM) [8]. It was consistent with apoptosis pattern found in our study for metastatic colon cancer cells. Similar but less pronounced late apoptosis was caused by TFN at 50 and 100 μM in TNBC cells after 48 hours [11]. The apoptotic TFN effect seems to be dependent on tumor type. As moderate early (10.44%; 8.46%) and late (5.69%; 9.78%) apoptosis was observed in renal and bladder cancer cells, respectively [27]. Conversely, a dramatic increase in late apoptosis (58.6%) was found in BE (2)-C neuroblastoma cells [28].

## 5 Summary

To the best of our knowledge it is a pioneer research on anticancer TNF potential at hypoxia and tissue normoxia i.e. oxygen concentrations present at tumor microenvironment. Since leflunomide has been used as an immunomodulatory and anti-inflammatory disease-modifying drug for a long period, its pharmacokinetic and pharmacodynamic data are comprehensively evaluated. Our study revealed that TFN at pharmacologically achievable concentrations effectively decreased tumor cell proliferation and viability at physiological oxygen levels. It should be noted that both primary and metastatic cancer cells are TFN sensitive. Moreover, hypoxia enhanced TFN cytotoxic effect confirming influence of oxygen tension on DHOH activity. Our *in vitro* evaluations of TFN action on primary and metastatic colon cancer cells have shown that leflunomide - non-toxic, widely used agent could be valuable addition to therapy. We are convinced that our mechanistic evidence will complement the knowledge about repurposing of leflunomide as a support treatment for colon cancer.

## Declaration of competing interest

The authors declare no conflict of interest.

Results were expressed as means ± SD.
